# SAPHO Syndrome: An Unusual Cause of Dysphagia

**DOI:** 10.5811/cpcem.2021.6.53001

**Published:** 2021-09-09

**Authors:** Megan Hoffer, Michaela Salvo, Sonal Batra

**Affiliations:** The George Washington School of Medicine and Health Sciences, Department of Emergency Medicine, Washington, District of Columbia

**Keywords:** dysphagia, SAPHO syndrome, emergency department

## Abstract

**Case Presentation:**

This case describes a 51-year-old male who presented to the emergency department with a complaint of two weeks of progressively worsening dysphagia as well as the emergence of superficial fluid collections on the anterior chest and leg during the same period. Computed tomography showed retropharyngeal and paratracheal fluid collections with adjacent vertebral osteitis; however, biopsies were negative for any infectious or mycobacterial source, and instead showed chronic inflammatory changes.

**Discussion:**

Synovitis, acne, pustulosis, hyperostosis, osteitis (SAPHO) syndrome is a rare rheumatic disorder that presents with multifocal osteitis and sterile neutrophilia. SAPHO syndrome may be easily mistaken for a diffuse infectious process on initial evaluation and imaging; however, it is treated with anti-inflammatory medications, including non-steroidal anti-inflammatory drugs and corticosteroids. Although most patients achieve remission of symptoms with treatment, the location of the fluid collections and resultant bony destruction may be life-threatening if undiagnosed.

## CASE PRESENTATION

**Image 1:**
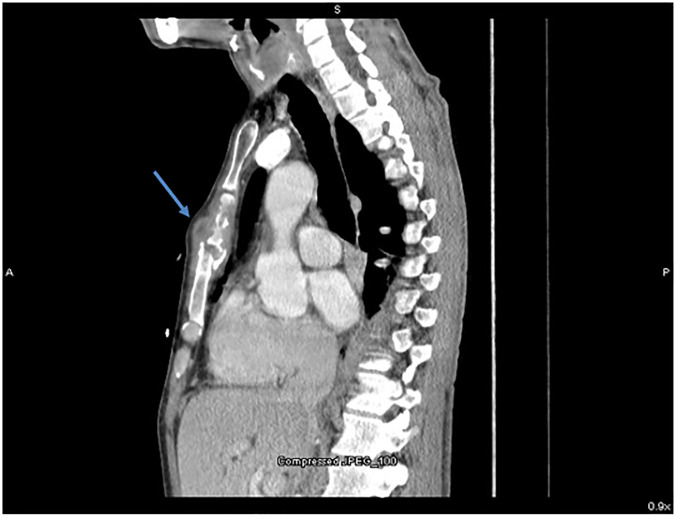
Computed tomography of the chest with contrast (sagittal view) showing fluid enhancing mass overlying the sternum with bony destruction of the sternum (blue arrow).

A 51-year-old male with a history of human immunodeficiency virus presented to the emergency department (ED) with two weeks of progressively worsening hoarseness and dysphagia. During the same period of time, he noted the development of two superficial fluctuant areas on the anterior chest and right leg. The patient denied any associated symptoms, including fever. He was born in Brazil and had a negative tuberculosis (TB) test in the United States. During the interview, he was notably hoarse but otherwise non-toxic in appearance. On examination, he had an area of fullness adjacent to the trachea, and a tender, fluctuant mass on the anterior chest. His lungs were clear to auscultation, no stridor was appreciated, and he had no pharyngeal erythema on inspection. He was afebrile, with oxygen saturation of 99% on room air with normal work of breathing and otherwise unremarkable vital signs. Laboratory workup was unremarkable, and computed tomography of the neck and thorax were obtained (Image 1).

## CASE DISCUSSION

**Image 2:**
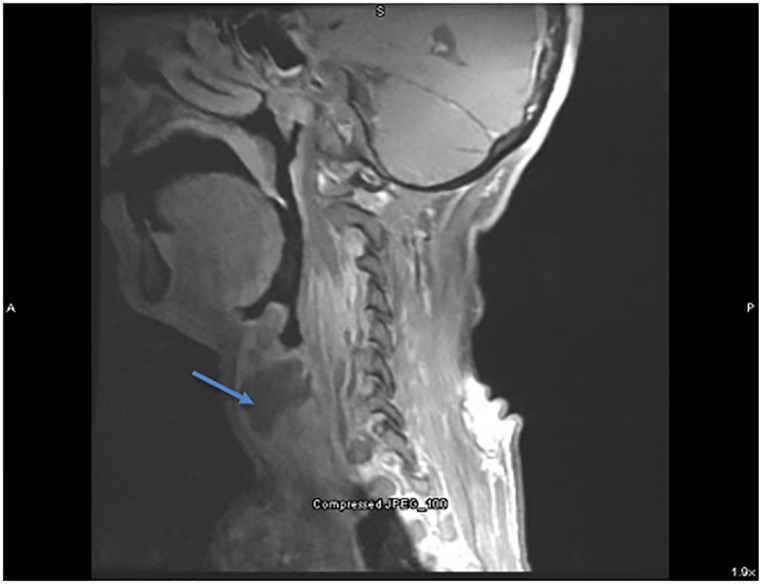
Magnetic resonance imaging of the neck (sagittal view) showing anterior fluid collection partially obstructing the trachea (blue arrow).

**Image 3:**
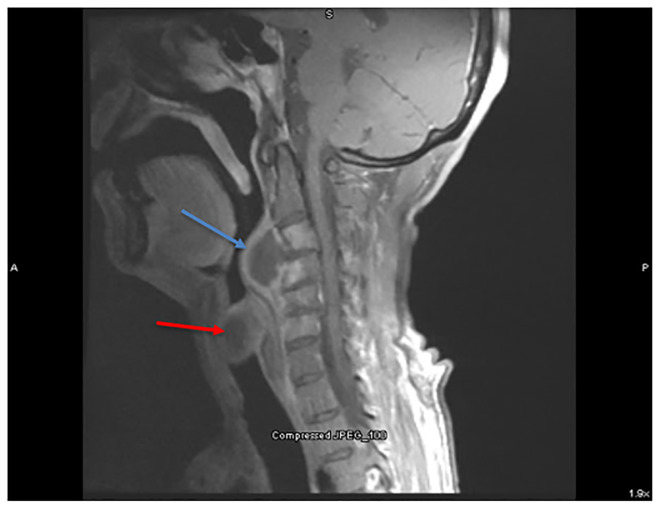
Magnetic resonance imaging of the neck (sagittal view) showing two more enhancing fluid collections, one retropharyngeally abutting the first and second cervical vertebrae (blue arrow), and the other extending into the trachea (red arrow).

Synovitis, acne, pustulosis, hyperostosis, osteitis (SAPHO) syndrome. Computed tomography showed multiple fluid collections in the soft tissues of the neck, chest, and lung, with bony destruction of the sternum and vertebrae. Magnetic resonance imaging showed retropharyngeal fluid collection with vertebral osteitis (Images 2–3). Otolaryngology was consulted in the ED, and the fluid collections were biopsied. Broad spectrum antibiotics were initiated, and he was admitted for further inpatient care and anticipated otolaryngology intervention. Although initially concerning for a diffuse infectious process, cultures of the fluid collections were negative and pathology showed chronic inflammatory changes with areas of necrosis. Serial TB tests, blood cultures, and an echocardiogram were also negative.

**CPC-EM Capsule**

What do we already know about this clinical entity?

*Synovitis, acne, pustulosis, hyperostosis, osteitis syndrome is a rare sterile neutrophilic disorder producing fluid-filled collections which may invade local structures.*

What is the major impact of the image(s)?

*Although the fluid collections may be benign, their location may result in airway compromise and destruction of local bony structures.*

How might this improve emergency medicine practice?

*The presence of a superficial fluid collection in conjunction with ‘red-flag’ symptoms such as dysphagia warrants imaging to identify lesions in high risk areas.*

This syndrome is a rare, sterile neutrophilic disorder that can be characterized by multifocal sterile osteitis, most often involving the anterior chest.^1^ The diagnosis is made clinically once infectious etiologies are excluded. One theory for the etiology of the disorder is an autoimmune reaction to the common *Propionibacterium acnes* bacteria.^2^ Treatment consists of non-steroidal anti-inflammatories, corticosteroids, tumor necrosis factor-alpha inhibitors, and bisphosphonates.^3^ Once the condition is identified, patients typically respond well to treatment with remission of symptoms.^4^ Following his admission, the patient underwent incision and drainage of the fluid collections adjacent to the trachea with otolaryngology, and recovered well with no complications. He was discharged home to continue outpatient care with rheumatology.
